# Repeated, noninvasive, high resolution spectral domain optical coherence tomography imaging of zebrafish embryos

**Published:** 2008-11-30

**Authors:** Larry Kagemann, Hiroshi Ishikawa, Jian Zou, Puwat Charukamnoetkanok, Gadi Wollstein, Kelly A. Townsend, Michelle L. Gabriele, Nathan Bahary, Xiangyun Wei, James G. Fujimoto, Joel S. Schuman

**Affiliations:** 1 University of Pittsburgh Medical Center Eye Center, Eye and Ear Institute, Ophthalmology and Visual Science Research Center, University of Pittsburgh School of Medicine, Pittsburgh, PA; 2 Department of Bioengineering, Swanson School of Engineering, University of Pittsburgh, Pittsburgh, PA; 3 Department of Medicine, Division of Oncology, Molecular Genetics and Biochemistry, McGowan Institute of Regenerative Medicine University of Pittsburgh School of Medicine, Pittsburgh, PA; 4 Department of Electrical Engineering and Computer Science and Research Laboratory of Electronics, Massachusetts Institute of Technology, Cambridge, MA

## Abstract

**Purpose:**

To demonstrate a new imaging method for high resolution spectral domain optical coherence tomography (SD-OCT) for small animal developmental imaging.

**Methods:**

Wildtype zebrafish that were 24, 48, 72, and 120 h post fertilization (hpf) and *nok* gene mutant (48 hpf) embryos were imaged in vivo. Three additional embryos were imaged twice, once at 72 hpf and again at 120 hpf. Images of the developing eye, brain, heart, whole body, proximal yolk sac, distal yolk sac, and tail were acquired. Three-dimensional OCT data sets (501×180 axial scans) were obtained as well as oversampled frames (8,100 axial scans) and repeated line scans (180 repeated frames). Scan volumes ranged from 750×750 µm to 3×3 mm, each 1.8 mm thick. Three-dimenstional data sets allowed construction of C-mode slabs of the embryo.

**Results:**

SD-OCT provided ultra-high resolution visualization of the eye, brain, heart, ear, and spine of the developing embryo as early as 24 hpf, and allowed development to be documented in each of these organ systems in consecutive sessions. Repeated line scanning with averaging optimized the visualization of static and dynamic structures contained in SD-OCT images. Structural defects caused by a mutation in the *nok* gene were readily observed as impeded ocular development, and enlarged pericardial cavities.

**Conclusions:**

SD-OCT allowed noninvasive, in vivo, ultra-high resolution, high-speed imaging of zebrafish embryos in their native state. The ability to measure structural and functional features repeatedly on the same specimen, without the need to sacrifice, promises to be a powerful tool in small animal developmental imaging.

## Introduction

Zebrafish embryos (*Danio rerio*) have gained popularity as a model for the study of the effects of genetic mutations on the structural development of the eye, as well as the brain, heart, spine, and gut [1-16]. Detailed study of the internal structures of the developing zebrafish embryo frequently requires either the sacrifice of the animal, or introduction of fluorescing molecules [17-19]. Confocal microscopy is useful in the visualization of fine structure; however measurements from images acquired with a microscope system require spatial calibration, and processing of microscopy video image sequences requires labor and time intensive video frame grabbing and analysis [20]. Multiphoton fluorescence microscopy allows three-dimensional (3D) imaging of cellular structures [21,22] but requires transcription of cDNA for fluorescent proteins into the animal, potentially altering the model [23,24].

3D microscopy can provide a comprehensive description in morphological studies [25]. Previous studies have also demonstrated the ability of optical coherence tomography (OCT) to create cross-sectional images through *Xenopus laevis*, *Rana pipiens*, and *Brachydanio rerio* embryos [26,27]. However the ability of a single line scan image to describe and assess complex 3D structures is limited. Moreover, single line scan images with conventional time domain OCT provide information along one scanning plane only, without the ability to visualize any other location. Doppler spectral domain OCT has been used to localize moving scattering media with *Danio rerio*; however the ability of spectral OCT to provide 3D structural assessments has yet to be explored [28]. Doppler OCT imaging, using time domain detection techniques, has been demonstrated for investigating cardiac dynamics in *Xenopus laevis* with 13.5 µm axial resolution in air, approximately 10 µm in tissue, at 1.3 µm wavelengths with 8,000 Hz axial scans (A-scans) per second [29]. Gated embryonic cardiography was demonstrated for 3D OCT imaging of the chick and mouse hearts using time domain detection with 14 μm axial resolution in air, an estimated 10 µm in tissue at 4,000 axial scans (A-scans) per second [30]. Spectral domain OCT with 2 μm axial resolution in tissue was demonstrated using a broadband titanium sapphire femtosecond laser centered at 800 nm using time domain detection at 40 axial scans per second and spectral/Fourier domain detection at 29,000 axial scans per second [31]. The high acquisition speeds enabled 3D OCT imaging of the developing embryonic murine cardiovascular system. High-speed time domain OCT acquiring 12,950 axial scans per second and swept source/Fourier domain detection with 25 images of 512 axial scans per second have been used to investigate Doppler and 3D OCT in the developing *Xenopus laevis* [32]. Very high-speed OCT using swept source/Fourier domain imaging was recently demonstrated at 7 µm axial resolution in tissue with a 1.3 μm wavelengths at 100,000 axial scans per second using Fourier domain mode-locked lasers [33].

In this investigation we apply high resolution spectral domain optical coherence tomography (SD-OCT) using spectral/Fourier domain detection which achieves 3.5 µm axial resolution with 800 nm light and 24,000 axial scans (A-scans) per second [34,35]. Rapid data acquisition combined with an improved axial resolution yields an imaging platform capable of the rapid acquisition of 3D data sets and repeated line scans suitable for averaging. Imaging was performed in the developing zebrafish embryo eye, an important genetic model system. The purpose of the present study was threefold: 1) to demonstrate the ability of SD-OCT to create and visualize virtual 3D data sets and known structures within zebrafish embryos at 24, 28, 72, and 120 h postfertilization (hpf) with comparison to histological section; 2) to demonstrate SD-OCT’s ability to visualize and measure developmental changes between cohort groups of embryos at 24, 28, 72, and 120 hpf, and to demonstrate the ability to make longitudinal observations across multiple days within a single embryo; and 3) to demonstrate SD-OCT’s ability to identify tissue-specific pathologies associated with genetic mutation.

## Methods

Animal care guidelines comparable to those published by the Institute for Laboratory Animal Research (Guide for the Care and Use of Laboratory Animals) and the US Public Health Service (Public Health Service Policy on Humane Care and Use of Laboratory Animals) were followed. This study was approved by the Institutional Animal Care and Use Committee (IACUC) of the University of Pittsburgh.

### Zebrafish care and the preparation for imaging

**Figure 1:**
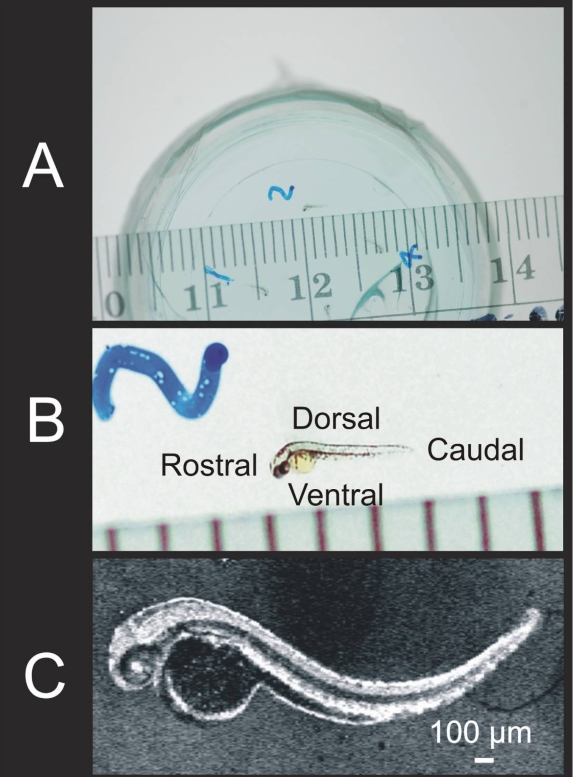
The appearance of the 72 hpf zebrafish with millimeter ruler, at magnification, and as observed by SD-OCT provided for appreciation of its small size. Zebrafish embryos were embedded in 1% agarose gel in an inverted microscopy Petri dish (**A, B; B** shows a magnified region of **A**). Embryos were scanned in three dimensions (3D), and reflectance of internal structures quantified. C-mode sections of the 3D data set could be isolated and tissue reflectance within a slice is displayed (**C**).

AB wildtype adult fish was raised in the main fish system of University of Pittsburgh. Before the experiment, a female fish was mated with a male. The mated fish produced embryos the following morning. Embryos were collected closely after labor in order to document the exact time of fertilization. For example, if an embryo was laid at 9:00 AM, 9:00 AM the next day was considered to be 24 hpf. Embryos were incubated at 28.5 °C in E3 egg water until desired developmental stage was reached. At the desired stage, the chorion was removed with forceps, and the embryo was embedded in 1% low melting agarose for imaging. E3 egg water is an ionic solution designed for zebrafish embryo cultures. The recipe used in the present study was: 5 mM NaCl, 0.17 mM KCl, 0.33 mM CaCl_2_, 0.33 mM MgSO_4_, and 0.1% methylene blue. Three imaging preparations were used in this study. Preparation 1: AB wildtype zebrafish embryos were incubated at 28.5 °C in E3 egg water until desired developmental stages (24, 48, 72, or 120 hpf). In this preparation, embryos were imaged only once, and then sacrificed. Four embryos were embedded in 1% low melting agarose (Fisher Scientific, Pittsburgh, PA) in a FluoroDish (World Precision Instrument, Sarasota, FL) at each developmental stage (Figure 1). The embedded embryos were then immersed in E3 egg water for live SD-OCT imaging. After SD-OCT imaging, fish were removed from the agarose gel and were fixed immediately for histological sectioning. Preparation 2: Three embryos were embedded in agarose gel as described in the previous preparation, imaged at 72 hpf, returned to E3 egg water, and then reembedded in agarose and imaged again at 120 hpf. Preparation 3: Three *nagie oko* (m520 allele) mutant embryos were embedded in agarose as described in Preparation 1, and imaged at 72 hpf [36].

### SD-OCT imaging

SD-OCT using spectral/Fourier domain detection technology has been described previously [34,35]. Briefly, light from a wide bandwidth low coherence source (Superlum Broadlighter, Moscow USSR; T840, 100 nm full width half maximum bandwidth centered at 840 nm) was split in a 50:50 coupler, and ported to a reference mirror and zebrafish embryos, respectively. Backscattered light from each were coupled, and the resulting interference pattern quantified by a linear array detector (E2V Aviiva camera, 2,048 pixels, each 14 µm wide). Unlike time domain OCT, which acquires A-scans pointwise at individual spatial depth locations using a scanning reference mirror, spectral/Fourier domain OCT uses a stationary reference mirror and obtains an entire A-scan depth profile instantaneously (50 μS).

OCT generates in vivo cross-sectional images of the target tissue using a near infrared light source, and measuring the echo time delay and magnitude of light, analogous to ultrasound imaging, though ultrasound is direct measurement while time of flight in OCT is measured by interferometry. While ultrasound requires an acoustic transduction medium (usually gel or saline solution), OCT permits noninvasive, noncontact imaging. Fourier domain detection has a high scanning rate, usually between 20,000 and 25,000Hz, allowing 3D scanning. The SD-OCT system used in this study is a prototype device that provides 3.5 μm axial resolution that is roughly 2–3 times higher resolution than the conventional time domain instruments used in ophthalmology, and approximately twice the resolution of currently available commercial Fourier domain OCT devices.

### Imaging protocols

**Figure 2:**
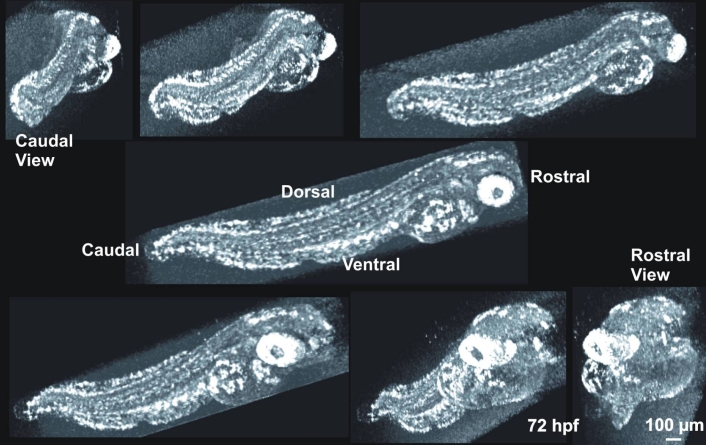
Rotation of a 3D image dataset of a 120 hpf embryo. SD-OCT can acquire a 3 dimensional quantitative description of the tissues within zebrafish embryos. These data allow the non-invasive visualization of the entire animal, as well as cross-sectional slices in any orientation through the animal at any stage of development, all without sacrificing the animal or noticeably impeding development. This gross anatomical visualization of the zebrafish was rendered in 3D-View, using the maximum intensity projection display.

Petri dishes were suspended vertically for SD-OCT imaging (Figure 1). Next, 1.5 mm×1.5 mm×1.4 mm body-centered 3D data volumes were obtained at a scanning density of 501×180×1024 pixels in 3.8 s (Figure 2). In the 120 hpf fish, it was necessary to increase the scan volume size to 3 mm×3 mm×1.4 mm to image the entire fish. Two 3D volumes with high transverse scan density with dimensions of 750 μm×750 μm×1,400 μm were also obtained, centered on the eye and heart, respectively. Repeated transverse plane line scans were obtained at 47 frames per second. For each scan, 180 frames were obtained. Each frame contained 501 transverse pixels along 750 μm length, with 1024 pixels in depth of 1,400 μm. Line scans were centered on the eye, ear, midbrain, heart, upper yolk sac, midyolk sac, lower yolk sac, and tail. In the 72 and 120 hpf fish, multiple line scans were obtained in a series of transverse scans from superior to inferior across the heart and pericardial cavity. Repeated line scan frames were acquired at 47 per second (180 sequential frames, each consisting of 501 A-scans acquired at 24,000 A-scans per second), providing a video image of the beating heart. After SD-OCT imaging, excluding the three embryos imaged over time on separate days, embryos were immediately fixed for histological processing and sectioning. Observed structures were compared to histology.

### Image processing

Stereo 3D image pairs were created to display 3D data sets using 3D View software (3D View v1.2, 2004; RMR Systems Ltd., Suffolk, UK; Figure 2). 3D data sets were pre-processed in Image J (v1.37, National Institutes of Health, Bethesda, MD). Specifically, window and level were adjusted to minimize the visibility of the surrounding agarose gel, as well as to maximize the visibility of the embryonic structures. Image size was reduced to 256 pixels×250 pixels×180 pixels to facilitate 3D processing. After creation of the 3D image pairs, contrast was optimized in Corel PhotoPaint (v12, Corel Corporation, Ottawa, Ontario, Canada).

Two-dimensional slabs of pixels sampled from the 3D data set, C-mode sections, were oriented the sagittal plane in the present study (Figure 1C). Reflectance within the sample tissue was summed and displayed. The thickness, location, and shape of the slice could be adjusted.

Three different averaging strategies were used to improve visualization of various structures:

#### Averaging frames containing different tissue over a small separation in space

**Figure 3:**
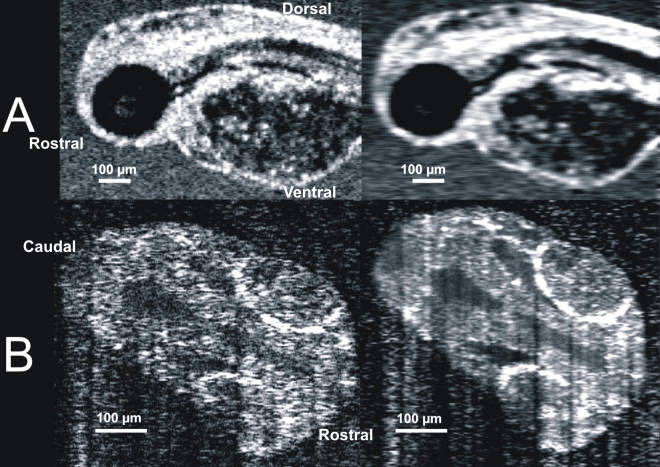
Data averaging reduces noise and enhances contrast between structures. **A:** A rolling 3-frame average within a 120 h post fertilization (hpf) embryo improves visualization of soft tissue structures in the gut while blurring the sharp features of the eye and ear. **B:** A side by side comparison of a single frame and a 120 frame averaged within the brain of a 24 hpf embryo demonstrates that aggressive averaging of repeated line scans increased visualization of soft tissue features and reduced noise.

A smoothing filter enhanced visualization of soft tissue features within the 3D data set (Figure 3A). 3D data smoothing was accomplished using a rolling 3-frame average. For example, the new frame 2 was an average of original frames 1–3; the new frame 3 was an average of original frames 2–4, etc.

#### Averaging a large number of frames containing the same tissue: averaging of a large number of frames was applied to repeated line scans of stationary tissue

Any number of frames could be averaged; however, most repeated line scan averaged images used between 60 and 120 frames (1.25 and 2.5 s long frames, respectively; Figure 3B).

#### Averaging frames containing moving tissue over a short length of time

A localized averaging filter was available for repeated line scans of moving tissue—specifically, the beating heart. The same location contributes to the averaged image; however, 3 images acquired at a 47 Hz sampling rate are used for averaging. These images span a 0.067 s interval of time. Any tissue movement that occurs within that time contributes to image degradation.

### JB4 embedding and sectioning

Following the final SD-OCT scanning, the embryos were fixed in 4% paraformaldehyde in 1X phosphate buffered saline (137mM NaCl, 2.7mM KCl, 4.3mM Na_2_HPO_4_, 1.47mM g KH_2_PO_4_, resulting in a pH of 7.4) at room temperature overnight, then dehydrated and embedded in JB4 resin (Polysciences, Inc., Warrington, PA) following the manufacturer’s protocol. The embedded embryos were sectioned with a Shandon Finesse microtome (Thermo Fisher Scientific, Inc., Waltham, MA) at 4 μm, stained with 1% methylene blue:1% azure II and observed and photographed with an AH2 Olympus microscope (Olympus America Inc., Center Valley, PA) and a WICAM camera (Qimaging, Surrey, BC, Canada).

### Quantitative tissue measurements

**Figure 4:**
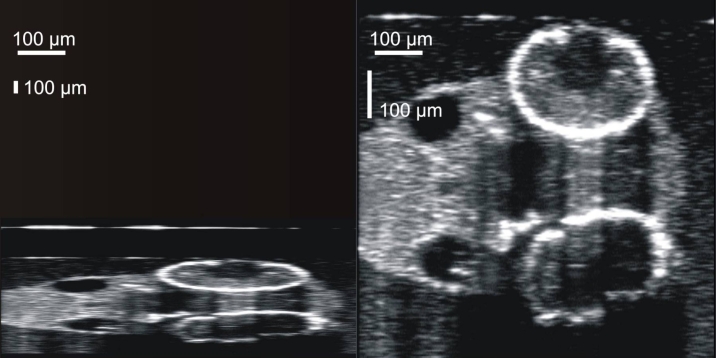
Images were resampled to correct the irregular data density of the raw scans. The Z axis (vertical axis of the image) is fixed at 1,024 reflectance measurements along a 2 mm line.  A X,Y scanning pattern was optimized for the body of the animal, with a X,Y a-scan density of 501x180, yielding the uncorrected aspect ratio observed in the left frame.  Scan data were resampled, utilizing spline interpolation, to produce images with a 1:1 aspect ratio for structural measurements and visualization.

The depth or z-axis scan length of all anatomic slices was 1,400 μm. Transverse scan lengths varied from 750 μm to 4,000 μm. Regardless of the physical scan size, all anatomic images were displayed with 501 pixels vertically and 1,024 horizontally. To perform anatomic measurements, we created a 1:1 aspect ratio by resampling images in the vertical direction (Figure 4). Tissue structures within SD-OCT and histological images were quantified using measurement calipers in CorelDRAW (v12, Corel Corp.). Distances were measured in pixels, and the lengths converted to real units based on the known image dimensions (Figure 4). Each tissue location was independently measured three times, and the average and standard deviation calculated. A single observer performed all measurements. Measurements of retinal thickness were acquired along the optical axis from the inner limiting membrane to the retinal pigment epithelial layer and choriocapillaris complex (RPE/CC). Measurements of the size of the pumping heart were obtained from averaged repeated line scans (60 to 120 frames averaged, as described in the previous section) containing at least 4 cardiac cycles. The longest distance across both chambers of the heart was measured.

### Statistics

The effect of age on measurements of retinal thickness and heart diameter at 24, 48, 72, and 120 hpf were compared by Fischer’s protected least significant difference Analysis of Variance. Individual levels of significance were determined by post-hoc Student’s *t*-test. Histological section measurements and SD-OCT retinal thickness measurements were compared by paired Student’s *t*-test. The *t*-test p-values were adjusted by Bonferroni’s method to account for multiple comparisons. P values of 0.05 or less were considered statistically significant.

## Results

### Visualization

**Figure 5:**
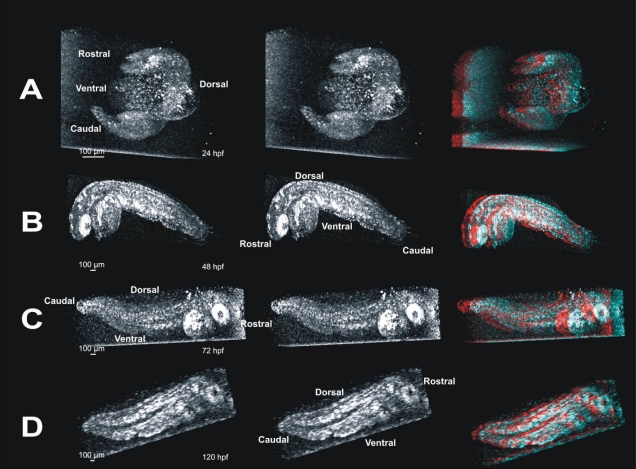
Stereo pairs of images reveal internal structures in whole embryos in three dimensions. These panels provide visualization of three dimensional (3D) data in a printed image.  Such data are best visualized interactively, rotating the projected data manually to obtain an optimal view of the structure of interest; impossible in printed image (see also Figure 2).  Panel **A** contains a 3D crossed-stereo image of a 24 hpf embryo. To view, gently cross your eyes until 3 images appear, and focus on the image in the middle. The image on the far right of panel **A** is the same image, and can be viewed with red/blue 3D glasses to visualize the embryo in 3D. Panel **B** contains a crossed-stereo pair and red/blue stereo image of a 24 hpf embryo. Panel **C** contains a 72 hpf stereo-pair and red/blue 3D image. In panels **A**-**C**, the embryo appears to be facing out of the image. Panel **D** contains a stereo-pair and red/blue 3D image of a 120 hpf embryo facing into the image.

Four embryos each were successfully imaged at four different stages of development: 24 hpf, 48 hpf, 72 hpf, and 120 hpf. 3D data sets were assembled from raster cube scans and could be observed from any angle, providing detailed and arbitrary visualization of the embryo (Figure 2). Utilization of stereo pairs allowed visualization of the virtual embryo in a 3D space (Figure 5).

**Figure 6:**
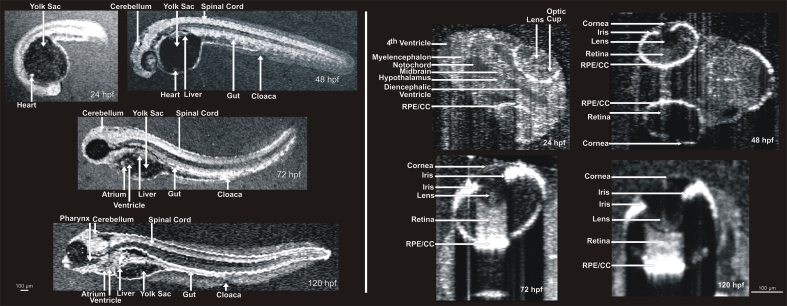
Visualization of developing internal anatomy of zebrafish embryos. Twenty-four hours post fertilization (hpf): A C-mode slice (left, 750 μm) centered on the eye, and a repeated line scan (right, 750 μm) centered on the brain and eye provide visualization of numerous internal structures within the brain, eye, and gut, as well as fluid spaces developing as ventricles within the brain. 48 hpf: C-mode slices at the level of the ear (left, 750 μm) and notochord (right, 1.5 mm). Microscopic structures of the eye, brain, gut, and heart can be visualized throughout. Blood within vessels and the heart create a bright reflection when isolated in a sagittal plane (left) while casting shadows on underlying tissues (right). 72 hpf: An averaged repeated line scan of the eye (left, 750 μm) reveals the cornea, lens, retina, and retinal pigment epithelial and choriocapillaris complex (RPE/CC) layer of the right eye of a zebrafish embryo. The RPE/CC of the left eye is the only structure within the left eye with sufficient reflectance to be observed. The C-mode slice (right, 1.5 mm) centered on the heart provides visualization of both chambers of the heart as well as numerous structures within the gut and brain. 120 hpf: An averaged repeated line scan of the eye (left, 750 μm) shows the cornea, lens, retina, and RPE/CC layer of the right eye. A C-mode slice (right, 4 mm) displays structures of the heart, gut, and brain, documenting the development that has occurred in only 120 hpf.

**Figure 7:**
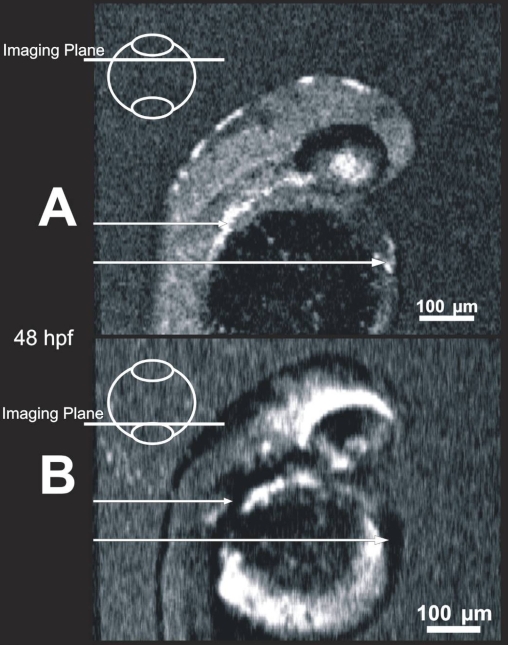
Blood within vessels creates shadow artifacts in C-mode slices below the vessels. Images of the same 48 hpf embryo obtained in a shallow slab location (**A**) and a deep location (**B**) allow visualization of blood, which is highly scattering (**A**, bright locations, arrows), and the resultant shadows mask structure in deeper slabs (**B**, dark locations, arrows).

Numerous internal structures could be visualized and measured within embryos at each age (Figure 6). Specifically, averaged repeated line scans provided C-mode sections of the eye, brain, heart, and gut at 24, 48, 72, and 120 hpf. Comparison with published anatomic sections was used to identify numerous organ structures within the SD-OCT cross-sectional images at ZFIN. Neural structures included the cerebellum, midbrain, hindbrain, otolith, spine, and notochord. Visualized ocular structures included the cornea, anterior chamber, iris, lens, vitreous, retina, and RPE/CC. The laminar structures of the ocular tissues could be discerned. By 72 hpf, the development of the iris pigment epithelium, the RPE/CC and the sclera had reached sufficient maturity to cast shadows, obscuring visualization of underlying structures. In these embryos, the retina was only visible when imaging through the pupil (Figure 6). At 24 hpf, the blood within the heart was visible as a bright concentrated reflective source (Figure 6). By 48, 72, and 120 hpf, cardiac tissue provided a significantly strong signal to visualize the walls of the heart. The heart appeared by SD-OCT as a single chamber at 24 and 48 hpf, and as two distinct chambers at 72 and 120 hpf. Visible structures within the body of the embryos included, but were not limited to, the liver, pharynx, swim bladder, gut, and anus (Figure 6). When C-mode sections were obtained from distal layers (relative to the location of scanning beam), shadows from blood in more proximal tissue could be misinterpreted as spaces in the deeper tissues (Figure 7).

**Figure 8:**
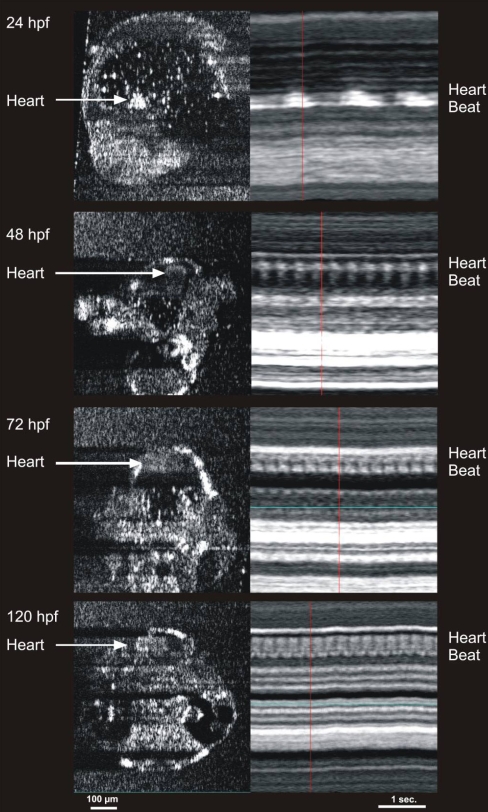
Cardiac M-mode images of the heart in 24, 48, 72, and 120 hours post fertilization embryos. The bright signal was created by blood within the heart. Note the increase in heart rate with development, as well as the development of two chambers at 72 hours post fertilization (hpf). The heart rates observed in the m-mode images are 47 beats per min (bpm) in the 24 hpf embryo, 157 bpm in the 48 hpf embryo, 219 bpm in the 72 hpf embryo, and 250 bpm in the 120 hpf embryo.

Visualization of the beating heart was accomplished at each stage of development (Figure 8). The eye, ear, beating heart, and spine were visualized and assessed in each animal. The gut was also visualized in fish by at least 48 hpf. Motion can be visualized using a video format. M-mode imaging (an A-scan acquired repeatedly for several seconds) through the heart allowed visualization and calculation of the heart rate (Figure 8). As heart volume increased, the blood within it provided sequentially brighter sources of reflection.

### Development and measurements

SD-OCT measurements of heart size and retinal thickness increased significantly with age (p<0.0001). Individual comparisons revealed a significance level of less than 0.0001 except for heart size between 72 hpf and 120 hpf (p=0.048) and retinal thickness between 24 hpf and 48 hpf (p=0.0002). Development from day to day was readily visible in the 3D virtual embryos and C-mode slabs (Figure 5 and Figure 6).

**Figure 9:**
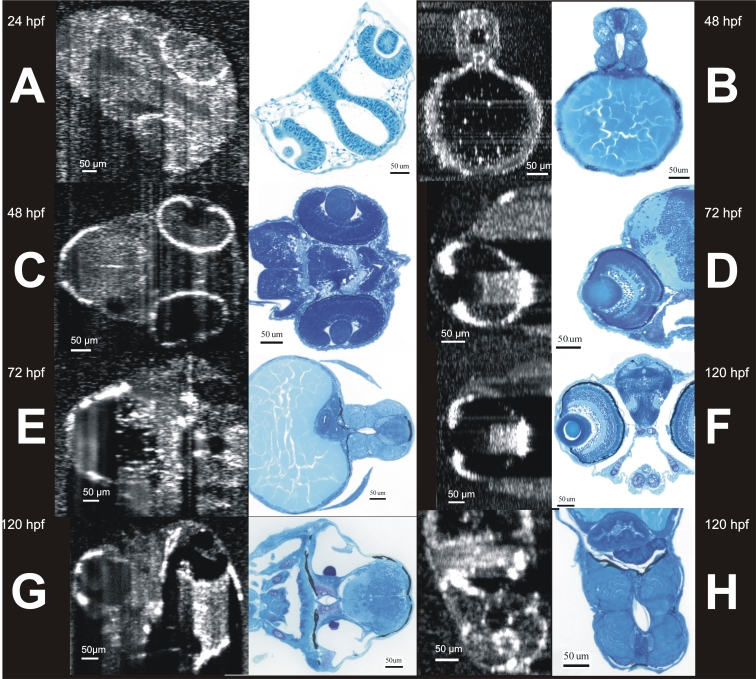
Side to side comparison of head and body structures visualized by SD-OCT and histology. SD-OCT images (left in each pair) were obtained noninvasively from living embryos, leaving them healthy and capable of continued growth. Similar structural data was obtained histologically, requiring sacrifice and sectioning before any information was obtained, and guaranteeing that any interesting structural observations can never be followed longitudinally. **A** shows the development of the eyes and ventricle of a 24 hours post fertilization (hpf) embryo. **B** and **C** show development of the spine and eyes of a 48 hpf embryo respectively. **D** and **E** show the development of the eye, spine, and liver at 72 hpf. **F**, **G,** and **H** show the development of the eye, ear, heart, and spine of the 120 hpf embryo.

There was excellent subjective correspondence between SD-OCT and histological sections (Figure 9). SD-OCT and histological retinal measurements are listed in Table 1. SD-OCT and histological measurements of retinal thickness all increased significantly with age (p<0.001). When all hpf ages were pooled, SD-OCT measurements of retinal thickness were significantly larger than histological measurements (p<0.0001, Table 1). When separated by age, mean SD-OCT measurements were all larger than histological measurements (Table 1).

**Table 1:** Comparison of optical and histological structural measurements.

**Hours post fertilization**	**SD-OCT retina (μm)**	**Histology retina (μm)**	**p**
Mean	85.81±27.18	66.06±19.17	**<0.0001**
24	51.78±8.74	38.95±2.45	**0.0055**
48	73.30±5.64	50.23±2.69	ns
72	108.86±8.07	79.26±9.70	ns
120	114.23±7.78	84.91±8.00*	**0.0015**

Measurements of retinal thickness (mean±SD) were obtained from SD-OCT and histological sections and compared using paired Student’s *t*-test. Significant values are given in bold. To account for 5 comparisons, Bonferroni’s correction was used to require an adjusted p value of 0.01 for differences to be considered statistically significant. Measurements of retinal thickness obtained from SD-OCT images in vivo were consistently thicker than those obtained from histological sections in vitro*.* The abbreviation “ns” stands for nonsignificant. The asterisk indicates that histological retinal sections at 120 hpf may have experienced some flattening.

At 24 hpf, an averaged line scan revealed development of the brain ventricles between the eyes, corresponding to the histological section of the same region (Figure 9A). A comparison of a SD-OCT scan and histological section of the 48 hpf distal yolk slice had excellent agreement between size and the position of the spine and gut (Figure 9B). As compared to eyes at the early stage of development, at 72 hpf prominent shadows were observed in the SD-OCT cross-sectional image cast by the iris pigment epithelium and the RPE/CC (Figure 9C-D). Blood within large vasculature also created shadows in SD-OCT images as could be observed in the yolk slice (Figure 9E). The excellent agreement between histological sections and SD-OCT was maintained through 120 hpf (Figure 9F-H).

### Longitudinal imaging in individual embryos

**Figure 10:**
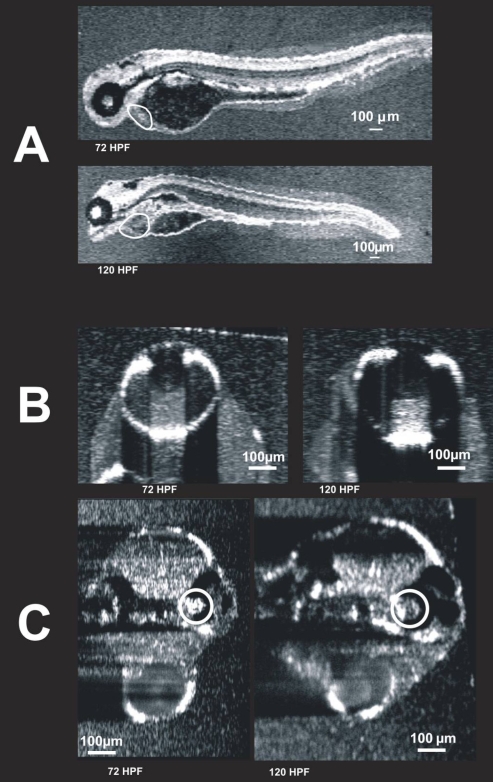
Visualization of individual animals imaged on two different days. These images were obtained from the same embryo on two different days: 72 hpf and 120 hpf. C-mode images of the heart (**A**, circled), eye (**B**), and ear (**C**, circled) are presented. The heart is also visible in **C**, but blurred due to averaging over multiple cardiac cycles. It is possible that the first imaging session altered development. To compare the 120 hpf twice-imaged embryos to 120 hpf embryos imaged only once, refer to Figure 6 and Figure 9.

All three embryos undergoing multisession (preparation 2) follow-up imaging were successfully removed from the agarose gel after imaging at 72 hpf. These embryos were reembedded and imaged at 120 hpf. Survival was confirmed by observable development in each of the embryos and beating hearts at 120 hpf. There was a readily observable increase in the length of the embryo between 72 and 120 hpf (Figure 10A). Ocular development was characterized by increased shadowing due to increasing light scattering associated with the development of the iris pigment epithelium, RPE/CC, and sclera (Figure 10B). The size of the optic cavity also increased with age. The size of both the swim bladder and pericardial cavities increased (Figure 10C). Development of the heart was characterized by increased size, and less empty space between the heart and the pericardial wall. Structures in embryos from preparation 2, i.e., those embryos imaged sequentially at 72 and 120 hpf shown in Figure 10, can be compared to structures in embryos imaged only once—specifically, embryos imaged at 72 hpf displayed in Figure 6, and embryos imaged at 120 hpf displayed in Figure 9.

### Visualization of developmental defects in the *nok* m520 mutant embryos

**Figure 11:**
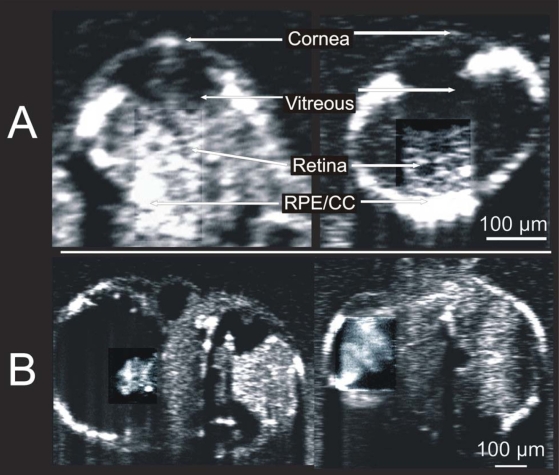
Visualization of mutations associated with *nok* m520 gene mutation. Side by side comparison of 72 hours post fertilization embryo eye (**A**) and heart (**B**) structures, with *nok* m520 mutant embryo genotypes on the left, and embryos of the same age without the *nok* m520 gene mutation. Contrast was enhanced in the retina and heart.

Mutations of the *nok* gene lead to developmental defects, as characterized by impeded development of the eye and heart, disorganization of the retinal layers, and an enlarged pericardial cavity [27]. Each of these characteristics was readily visualized in SD-OCT images of the mutant embryos (Figure 11). In the eye, expression of the m520 allele mutant *nok* gene presented with underdeveloped and disorganized retina and sclera (Figure 11A, left). By comparison, the normal 72 hpf retina is characterized by distinct vitreous, retina, and RPE/CC layers, and increased light absorption and scatter by more developed anterior segment structures (Figure 11A, right). In the heart, expression of the m520 allele mutant *nok* gene presented with an enlarged pericardial cavity and an underdeveloped heart (Figure 11B, left), compared to a normal 72 hpf heart and pericardial cavity (Figure 11B, right). This demonstrates that the SD-OCT technology is sufficiently sensitive to detect developmental defects at the microstructural tissue level.

## Discussion

SD-OCT provides visualization of numerous structures within the developing zebrafish embryo. Unlike fluorescence microscopy or histological sectioning, SD-OCT is noncontact, noninvasive, and does not require genetic or exogenous modification of the animal to identify substructure. As a result, SD-OCT allows multisession repeated imaging of individual embryos with no observable disturbance of development. We were able to qualitatively and quantitatively document development within living embryos. In the present study, there were no detectable effects on development observed at up to 120 hpf in embryos that had previously been embedded and imaged. Finally, we were able to noninvasively confirm the morphological defects of the *nok* mutation in 72 hpf embryos.

Development of highly scattering or absorbing tissues, such as the iris pigment epithelium, RPE/CC, and blood present a challenge in imaging deeper tissues using SD-OCT at near infrared wavelengths. By 72 hpf, it is difficult to visualize structures behind the eye, and by 120 hpf, those structures are completely shadowed. The visibility of structures behind light scattering or absorbing tissue is limited due to shadowing. For example, this is particularly well demonstrated due to blood in the heart, or due to pigment stripes in the skin of the developing fish (Figure 7). The use of longer wavelengths improves penetration and imaging behind high-scatter tissues, but at the expense of lesser axial resolution.

There are several limitations associated with SD-OCT imaging. As with time-domain OCT, speckle noise may obscure the image (Figure 3B). Signal averaging is effective in increasing signal to noise ratio and reducing speckle noise, however acquisition of a sufficiently large number of redundant images for averaging increases scan time, as compared with a single tomogram. Long scan times increase the probability of motion artifact when scanning living tissue. This limitation has greatly limited the use of time domain OCT for 3D acquisition, though application of biphase modulation techniques may increase time-domain scan times [37]. In the present study, most of the embryos at each age exhibited tail movements during the 4 s scan time. There is a trade-off between tissue motion and redundant data acquisition for averaging.

One potentially effective approach to speckle reduction and signal to noise improvement is sequential collection of data to be averaged. For example, if 3 scans are to be averaged for the final display image, it is possible to collect 3 A-scans before moving the beam to the next location. At a 24,000 Hz axial scan rate, this requires only 0.000125 s per A-scan. This would increase a 512 A-scan 180-frame raster scan time from 3.8 to 11.4 s. The rapid scan rate of sequential A-scans would provide the ability to do averaging of scans without motion artifacts within averaged A-scans; however, there would be numerous motion artifacts within the raster scan data set, as expected from any image acquired over 11 s in living tissue.

Retinal thicknesses measured by SD-OCT were consistently higher than those measured in corresponding histological sections (Table 1). When pooled together, and at two of the four time points, the difference reached statistical significance, though the consistency of the difference as well as the increasing magnitude of difference with time suggests a real and systematic difference between measurements of living tissue in situ and measurements of fixed histological sections. The difference between them increased steadily from 12 microns at 24 to 30 microns at 72 hpf, remaining at 30 microns at 120 hpf. The difference was highly significant when the measurements were pooled for all ages, and 2 of the 4 comparisons reached significance when separated by age. It is possible that tissue shrinkage was anisotropic due to the laminar structure of the retina [38]. Nonlinear tissue shrinkage has been a confounder in previous high resolution spectral domain OCT studies comparing tissue layers in animal models with histological sections [39,40]. It is likely that, in the present data set, the measurement differences were due, at least in part, to tissue shrinkage associated with dehydration and fixation. Typically, a correction factor of 15% is added to morphometric measurements of histological sections; however, shrinkage as great as 47% has been documented [41,42]. An average difference of 37.7% between SD-OCT and histological measurements of retinal thickness was observed in the present study.

In conclusion, SD-OCT is a promising technology providing the unique ability to perform completely noninvasive imaging of zebrafish embryos, an important model for studying gene expression. Acquisition of 3D data sets allows virtual anatomic sectioning of the embryo in any desired orientation. Due to its noninvasive nature, OCT can repeatedly image a single embryo from one day to the next with no observable effect on development. Furthermore, we have developed an imaging protocol that does not require anesthetizing agents, avoiding possible adverse effects on the embryos. This will improve the efficiency of studies since it reduces the need to sacrifice specimens, and significantly fewer specimens are required to obtain information on longitudinal changes. Further, assessment of a given animal over a period of time is likely to provide more accurate and useful longitudinal data than evaluation of populations of animals at different stages of development. Challenges remain in the reduction of noise and the optimization of signal. As with the introduction of imaging modalities in the past, improvements in image processing promise to increase the utility of this technique in the noninvasive and noncontact study of small animals, such as the zebrafish embryos shown in this investigation.
